# Genetic diversity of *Mycobacterium tuberculosis *Complex in Jos, Nigeria

**DOI:** 10.1186/1471-2334-10-189

**Published:** 2010-06-26

**Authors:** Agatha Ani, Torbjørn Bruvik, Yetunde Okoh, Patricia Agaba, Oche Agbaji, John Idoko, Ulf R Dahle

**Affiliations:** 1 Department of Medical Microbiology, Faculty of Medical Sciences, University of Jos, Nigeria; 2 Division of Infectious Disease Control, Norwegian Institute of Public Health, Oslo, Norway; 3 AIDS prevention initiative in Nigeria, Centre Jos University Teaching Hospital, Jos, Nigeria

## Abstract

**Background:**

Nigeria has a high tuberculosis incidence, and genotyping studies of *Mycobacterium tuberculosis *Complex (MTC) in the country are necessary in order to improve our understanding of the epidemic.

**Methods:**

Isolates of MTC were isolated from cases of pulmonary tuberculosis in Jos, North Central region of Nigeria during 2006-2008. Drug susceptibility test (DST) was performed on 77 of 111 isolates by proportion method on Lowenstein Jensen (LJ) slope while genotyping of mycobacterial DNA was performed by spoligotyping. The SpolDB4 database and the model-based program 'spotclust' were used to assign isolates to families, subfamilies and variants.

**Results:**

A total of 111 pulmonary isolates from consecutive tuberculosis patients in the city of Jos, Plateau State, Nigeria were spoligotyped. A total of 84 (76%) of the isolates belonged to the Latin American Mediterranean (LAM) family. Of these, 78 isolates were assigned to the LAM10 lineage. Among these, 66 exhibited identical spoligopatterns. Drug susceptibility profiles obtained were not consistently associated with any spoligopattern.

**Conclusions:**

The dominance of few *M. tuberculosis *lineages suggests either a high rate of transmission, frequent import of closely related strains, or a highly conserved genotype. It remains to be confirmed whether the predominance of identical LAM10 represent an outbreak.

Spoligotyping was useful to gain an overall understanding of the local TB epidemic. This study demonstrated that the incidence of TB in Jos, Nigeria may be caused by a few successful *M. tuberculosis *families, dominated by the LAM10 family.

## Background

Nigeria ranks fifth among the world's high-burden countries, with a number of tuberculosis (TB) cases of 450,000. The TB incidence is at 311/100,000 and the rate of new sputum smear positive disease is approximately 137/100,000 [1].

Published work on the incidence, drug susceptibility, and epidemiology of *Mycobacterium tuberculosis *Complex (MTC) in Jos and in Nigeria are scarce. The results of the few reported studies did not address epidemiological concerns [2-5]. Supporting data from additional studies are therefore needed to better understand the current epidemic in this area. Nigeria, like most nations of sub Saharan Africa has been rated endemic for TB due to the association of *Mycobacterium *species with the human immunodeficiency virus (HIV). It has been reported that 30-45% of HIV positive persons in Nigeria present with active TB at one point or the other [6].

Points of concern include the proportion of patients lost to follow-up, diagnostic delay, low case detection rate and the continuing high prevalence of HIV. The high case rate in many African countries contributed to a rise of the global TB incidence of 1% in 2003, despite stable or declining rates in the rest of the world. The incidence in Nigeria however, declined by 1.3% between 2005 and 2006 [1]. In order to improve our understanding of the TB epidemic in this high-incidence country, the current study included 111 strains of MTC species isolated from in Jos, Nigeria. For the genetic studies, DNA of all 111 isolates were obtained during three intervals of 2006-2008. Currently no laboratory in Nigeria offers spoligotyping services. Spoligotyping is a PCR-based fingerprinting method that detects the presence or absence of 43 defined spacers situated between short direct repeat (DR) sequences in the genomes of members of the MTC [7]. Important advantages of spoligotyping are that it is cheap, easy to perform and fast. In addition, it has been demonstrated that the results are highly reproducible [8]. Unique to spoligotyping results are tools like the SpolDB4 database [9] and the web-based computer algorithm 'Spotclust' [10] that can be used to assign new isolates to families, subfamilies and variants. The results from local studies can thus be analyzed and compared to the global MTC population. This may help to better understand the world-wide spread of common MTC families and subfamilies. In the current study we describe the diversity of MTC isolates from Jos, Nigeria, based on spoligotyping, and identify the families and subfamilies responsible for the current persistence and spread of TB in this high-incidence community.

## Methods

### Settings

Ethical clearance was granted respectively by The Jos University Teaching Hospital and Plateau State Hospital ethical committees. The study was descriptive of a bacterial collection and contained no material of human origin. Personal data were removed from all bacterial cultures to protect the anonymity of the patients. Patient informed consent was therefore not obtained.

### Sputum culture and Drug susceptibility test

Smear positive sputa from (93 new, 16 followup and 2 failed) cases of pulmonary TB were collected when available from the out patient department of 4 different TB-treatment centers in Jos. Specimens were processed for cultures of *Mycobacterium *species on Lowenstein Jensen (LJ) medium at the Department of Medical Microbiology, University of Jos, Nigeria as described previously [6].

A total of 77 of the 111 isolates that were phenotypically identified as MTC as previously described [5], were tested for drug susceptibility by the proportion method on LJ medium using 0.2 μg isoniazid (INH), 2 μg ethambutol (EMB), 40 μg rifampicin (RIF) and 4 μg streptomycin STR) [5].

### Spoligotyping

Cultures were heat inactivated at 80° for 20 minutes. A total of 111 heat-killed MTC strains were sent to the National Reference Laboratory for Mycobacteria at the Norwegian Institute of Public Health (NIPH). Upon arrival, DNA was extracted and spoligotyping performed as described elsewhere [7].

DNA was extracted and spoligotyping was performed according to Kamerbeek et al. [7]. A single linkage dendrogram was produced by use of the Bionumerics, version 5.1 software (Applied Maths, Kortrijk, Belgium).

### Family assignment

The obtained spoligopatterns were first compared to the SpolDB4 database and assigned to families and subfamilies [9]. Second, in order to assign names to the isolates not found in the SpolDB4 database, the spoligopatterns were analyzed with 'Spotclust', using a mixture model built on the SpolDB3 database [10]. This model takes into account knowledge of the evolution of the DR region and assigns spoligopatterns to families and subfamilies. The rate of diversity was calculated by dividing the number of spoligotypes by the number of isolates.

## Results

### Genetic diversity and family assignment

**Figure 1:**
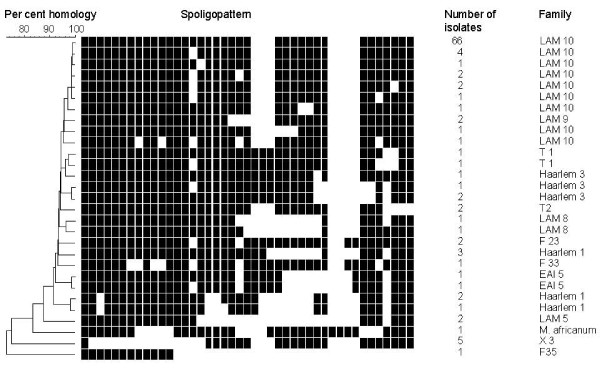
**Spoligopatterns and identity of *Mycobacterium tuberculosis *complex strains from Jos, Nigeria**. Genetic similarity and spoligopattern of *M. tuberculosis *Complex isolates from Jos in Nigeria. The population demonstrates a high degree of homogeneity and dominance of LAM10.

The 111 analyzed isolates gave 29 different spoligopatterns resulting in an overall diversity (number of spoligotypes divided by the number of isolates) of 27. A total of 17 spoligopatterns occurred only once and 1 pattern comprised 65% of the isolates (Figure 1). All patterns had been described previously [9]. Family assignment by use of the SpolDB4 database and 'Spotclust' showed that 84 (76%) of the isolates belonged to the Latin American Mediterranean (LAM) family, 79 (93%) of these belonged to the LAM 10 family. A total of 10 (9%) of the isolates were assigned to the Haarlem family, 6 (5%) to the X family, 4 (4%) to the T family, 4 (4%) to the F family, and 2 (2%) to the EAI family. One isolate was assigned to *M. africanum*. The low spoligotype diversity within the MTC population was confirmed by construction of a dendrogram. Most isolates demonstrated more than 90% homology, and only 6 isolates were less than 80% homologous to the other strains (Figure 1). The LAM10 clearly dominated the TB epidemic in Jos.

The abundance of the LAM family in this area may indicate that the family is either well adapted to spread, highly conserved, or recently transmitted within this community. The observation of only one isolate of *M. africanum *was unexpectedly low, as previous studies have demonstrated that approximately 13% of TB in humans in Ibadan, Nigeria was caused by *M. africanum *type I and *M. bovis *[2,3].

Drug susceptibility tests were available from 77/111 isolates in this study. A total of 31 strains were resistant to at least one drug, and 19 strains were multidrug resistant (resistant to at least rifampicin and isoniazid) (MDR) strains (Table 1). Although the numbers were low, it was noted that 16/56 tested LAM10 isolates were MDR and 3/4 tested Haarlem-family isolates were MDR.

**Table 1:** Spoligotypes and drug susceptibility patterns of *Mycobacterium tuberculosis *from Jos, Nigeria.

	No. of isolates	Assignment of *Mycobacterium tuberculosis *complex isolates						
		
		LAM 10	LAM	H	X	T, F	EAI	*M. africanum*
Total	111	79	5	10	6	4, 4	2	1
		(71%)	(4%)	(9%)	(5%)	(4%, 4%)	(2%)	(1%)

DST	77	56	4	4	4	4, 3	1	1

PS	46	31	3	2	2	4, 3	0	1

MDR	8	8	0	0	0	0, 0	0	0

MDR+	11	8	1	2	0	0, 0	0	0

Other	12	9	0	0	2	0, 0	1	0

Abbreviations: DST: Drug susceptibility test-results available, PS: Pan susceptible, MDR: Multidrug resistant, MDR+: MDR isolates with additional resistance, other: isolates resistant to drugs but sensitive to either rifampicin or isoniazid.

## Discussion

This study demonstrated that LAM10 isolates were abundant in the current population of MTC isolates from Jos, Nigeria. A study conducted in the West province of Cameroon found that 193 of 413 *M. tuberculosis *isolates belong to the Cameroon family (LAM10-CAM) [11], and in Harare, Zimbabwe, 68 of 214 isolates are LAM11-ZWE variants [12]. In Dar Es Salaam, Tanzania however, the overall genetic diversity was calculated at 52 and only 3 of 147 isolates were LAM 10 strains [13]. Of the 111 isolates in this study, 78 were LAM10 and no LAM11 isolates were observed. Although other lineages are found in other countries, these findings indicate that various LAM sub-families dominate in various regions of Africa and that TB epidemics are multiple and local. Other lineages that are abundant in other African countries (such as the LAM11) were absent in the current study. Thus, the MTC populations in high-incidence countries may vary greatly and can be difficult to estimate without molecular epidemiological studies.

The success of the LAM family in particular in this community is intriguing. The highly prevalent LAM10 family in this study may indicate that the family is spreading rapidly, but could also reflect a slow evolution of the DR region. However, one limitation of the current study is related to the uncertainty about the accurate representation of MTC population of Jos, Nigeria. Also, the lack of the more discriminatory methods such as, IS*6110 *RFLP and MIRU-VNTR typing [14-16], prevents more conclusive results.

The predominance of the LAM family in South America and West-Africa suggests a possible co-evolution between specific MTC families and host populations [9,17]. The molecular basis of this theory remains to be elucidated but this pattern might reflect the last centuries' transport and interaction between these continents and the genetic relatedness between Western Africa and the Americas.

The rates of drug susceptibility to first line anti tuberculosis drugs were evenly distributed among the genetic lineages in the current study (Table 1). This may indicate strain-strain heterogeneity or other factors which could not be explained within the scope of this study. Genetic fingerprinting methods with higher discriminatory tendencies are required for better interpretation of the results.

## Conclusions

Spoligotyping performed in this study was useful with insights on the local TB epidemic in Jos, Nigeria. This study demonstrated that the extensive TB epidemic in this area was caused by one successful *M. tuberculosis *family, dominated by the LAM10 subfamily. Import of new strains from neighboring countries appears to represent a minor problem, but such interpretation need confirmation. The spread of TB in this area may however be locally restricted.

## Competing interests

The authors declare that they have no competing interests.

## Authors' contributions

AA contributed to conception and design of the study, laboratory work and data analysis. TB, YO, PA, and OA participated in the clinical evaluation of the patients work and data analyses. JI participated in the design of the study and the data analyses. UD conceived the study, supervised and participated in the laboratory work and data analyses. All authors contributed in the writing of the article, read and approved the final manuscript.

## Pre-publication history

The pre-publication history for this paper can be accessed here:

http://www.biomedcentral.com/1471-2334/10/189/prepub
